# Switch/Sucrose Non-Fermentable (SWI/SNF) Complex—Partial Loss in Sinonasal Squamous Cell Carcinoma: A High-Grade Morphology Impact and Progression

**DOI:** 10.3390/cimb46110723

**Published:** 2024-10-30

**Authors:** Roberto Onner Cruz-Tapia, Ana María Cano-Valdez, Abelardo Meneses-García, Lorena Correa-Arzate, Adriana Molotla-Fragoso, Guillermo Villagómez-Olea, Diana Brisa Sevilla-Lizcano, Javier Portilla-Robertson

**Affiliations:** 1 Medical, Odontological and Health Science Doctorate Program, Oral and Maxillofacial Pathology Department, Postgraduate School of Dentistry, National Autonomous University of Mexico, Mexico City 04510, Mexico; onner-cruz@fo.odonto.unam.mx; 2 National Institute of Cancer, Postmortem Department, Head and Neck Pathology Service, Anatomic Pathology Division, Mexico City 14080, Mexico; acanova@incan.edu.mx; 3 National Institute of Cancer, Anatomic Pathology Division, Mexico City 14080, Mexico; aameneses@hotmail.com; 4 Epidemiology Department, University of the Valley of Mexico, Querétaro 76230, Mexico; lorena.correa@uvmnet.edu; 5 Oral and Maxillofacial Pathology Department, Postgraduate Dental School, National Autonomous University of Mexico, Mexico City 04510, Mexico; molo.22@fo.odonto.unam.mx; 6 Tissue Engineering Laboratory, Postgraduate Dental School, National Autonomous University of Mexico, Mexico City 04510, Mexico; guiviol@comunidad.unam.mx; 7 Immunohistochemical and Pathology Laboratory, Private Practice, Morelia 58260, Mexico; inmunodiagnostic@gmail.com

**Keywords:** sinonasal cancer, SMARCB1, SMARCA4, SWI/SNF-deficient tumors, squamous cell carcinoma

## Abstract

Sinonasal carcinomas are aggressive neoplasms that present a high morbidity and mortality rate with an unfavorable prognosis. This group of tumors exhibits morphological and genetic diversity. Genetic and epigenetic alterations in these neoplasms are the current targets for diagnosis and treatment. The most common type of cancer originating in the sinonasal tract is sinonasal squamous cell carcinomas (SNSCCs), which present different histological patterns and variable histological aggressiveness. A significant number of alterations have been reported in sinonasal tumors, including deficiencies in the Switch/Sucrose non-fermentable (SWI/SNF) chromatin remodeling complex. In the sinonasal tract, deficiencies of the subunits SMARCB1/INI1, SMARCA4/BRG1, and SMARCA2 have been noted in carcinomas, adenocarcinomas, and soft tissue tumors with a distinctive high-grade morphology and a fatal prognosis. Objective: The objective of this study is to identify the status of the SWI/SNF complex using immunohistochemistry in sinonasal squamous cell carcinomas and their association with morphology and survival. Methods: A total of 103 sinonasal carcinomas with different grades of squamous differentiation were analyzed; the selection was based on those cases with high-grade morphology. The carcinomas were then evaluated immunohistochemically for SMARCB1 and SMARCA4 proteins. Their expression was compared with the biological behavior and survival of the patients. Results: Among the SNSCCs, 47% corresponded to the non-keratinizing squamous cell carcinoma (NKSCC) type with high-grade characteristics, 40% were keratinizing squamous cell carcinomas (KSCCs), 9% were SMARCB1-deficient carcinomas, and 4% were SMARCA4-deficient carcinomas. Mosaic expression for SMARCB1 (NKSCC—33%; KSCC—21.9%) and SMARCA4 (NKSCC—14.6%; KSCC—12.2%) was identified, showing an impact on tumor size and progression. Conclusions: We identified that that the partial loss (mosaic expression) of SMARCB1 in SNSCCs is associated with high-grade malignant characteristics and a negative effect on patient survival; meanwhile, SMARCA4-mosaic expression in SNSCCs is associated with high-grade malignant characteristics and an increase in tumor size concerning the intact SMARCA4.

## 1. Introduction

The multiprotein Switch/Sucrose non-fermentable (SWI/SNF) complex, also known as BAF (BRG1-associated factor), in mammals consists of more than 20 subunits that vary by tissue type and embryonic stage. This complex plays a crucial role in gene expression and transcription regulation [1,2]. Deficiencies in specific core proteins that are required for the assembly of certain SWI/SNF subunits have been reported in different types of cancers, affecting multiple organs such as the lungs, genitourinary tract, gastrointestinal tract, central nervous system, and head and neck. The SWI/SNF chromatin remodeling complexes are responsible for the proper positioning of nucleosomes. SWI/SNF complexes belong to three subfamilies—canonical BAF (cBAF), GLTSCR1 and GBAF complexes, and polybromo-associated BAF (PBAF) [1,2].

Recently, a subset of carcinomas originating in the sinonasal tract has been identified with distinct biological aggressiveness, characterized by the complete loss of SWI/SNF complex subunits, particularly SMARCB1 (INI1) and SMARCA4 (BRG1)/SAMRCA2 [3,4]. Sinonasal carcinomas represent 4% of all malignant neoplasms affecting the head and neck, with the most common originating in the nasal cavity and paranasal sinuses [5]. SWI/SNF complex-deficient carcinomas are rare. However, this group includes carcinomas and adenocarcinomas that completely lose the expression of certain subunits of the complex [6]. In surgical pathology practice, the evaluation of the SWI/SNF complex has become a useful tool for the diagnosis and classification of neoplasms with an altered expression of complex subunits. In head and neck cancers, recent descriptions include neoplasms with deficiencies in certain subunits of the SWI/SNF complex, such as malignant rhabdoid tumors, head and neck epithelioid sarcoma, SMARCB1-deficient sinonasal carcinoma, SMARCB1-deficient sinonasal adenocarcinoma, SMARCA4-deficient sinonasal carcinoma, and SMARCA4-deficient sinonasal teratocarcinosarcoma [6].

Sinonasal squamous cell carcinoma (SNSCC) is the most frequent carcinoma that develops in the nasal cavity and paranasal sinuses. Its pathogenesis has been associated with exposure to wood dust, construction material particles, high-risk HPV infections, and precursor lesions such as sinonasal inverted papilloma—particularly the oncocytic variant [7].

SNSCCs are classified into two variants based on their histologic features and keratinization capacity. Keratinizing or conventional squamous cell carcinoma (KSSC) is characterized by aggressive squamous differentiation with evident keratin deposits and a diversity of growth patterns, including papillary, adenosquamous, basaloid, sarcomatoid, and acantholytic. Non-keratinizing squamous cell carcinoma (NKSSC) presents with a basaloid morphology, in absence or focal keratinization with a consistent transitional-type growth pattern. Sinonasal SCCs are associated with high morbidity and mortality due to their tendency to infiltrate extra facial and maxillary structures, as well as skull base invasion and ocular displacement due to the orbital extension [8,9].

The total or partial loss of expression of SWI/SNF complex subunits results from the inactivation of this chromatin remodeling complex, contributing to increased cytological aggressiveness and a poor prognosis in neoplasms [10]. Assessing the SWI/SNF chromatin remodeling complex has facilitated the classification and development of therapeutic strategies for various soft tissue tumors, carcinomas, and adenocarcinomas affecting organs such as the lungs, genitourinary tract, gastrointestinal tract, central nervous system, nasal cavity, and paranasal sinuses [3,4,11]. Immunohistochemical analysis has proven instrumental in demonstrating the total or partial loss of SMARCB1 (INI1) and SMARCA4 (BRG1) subunits, making it a valuable tool for diagnosis and therapeutic decision-making [12].

This study examines the expression of the SWI/SNF complex and its impact on the biological behavior of poorly differentiated or high-grade squamous carcinomas originating in the sinonasal tract. Through immunohistochemical analysis, we identified the inactivation or partial loss (mosaic expression) of SMARCB1 and SMARCA4 subunits, correlating with disease morphology and progression. These findings pave the way for future research to expand therapeutic options and refine the classification of this neoplasm.

## 2. Materials and Methods

### 2.1. Sample Selection

All cases diagnosed as sinonasal tract carcinomas at the National Cancer Institute of Mexico (INCan) were reviewed to identify and select squamous cell carcinomas and poorly differentiated carcinomas originating in the nasal cavity and paranasal sinuses, during the period from January 2006 to December 2018. Out of a total of 1582 cases of carcinomas originating in the sinonasal tract recorded over a 12-year period, only 103 were selected for inclusion in this study. The variables considered for the inclusion of samples were gender, age, tumor epicenter location, tumor size, histological diagnosis, presence of metastasis, oncological stage, and time elapsed from diagnosis to death or recurrence.

### 2.2. Morphologic Analysis and Tissue Microarray (TMA)

The available histological material for each case was reviewed with the aim of confirming the correct histological classification of the samples according to diagnostic criteria (5). Three study groups were identified: NKSCC (*n* = 48), KSCC (*n* = 41), and basaloid/poorly differentiated carcinomas (*n* = 14). The histological parameters evaluated in all groups included cytological morphology, growth pattern, presence of desmoplasia, neural and vascular infiltration, type and disposition of the inflammatory infiltrate, and necrosis. For the poorly differentiated carcinoma group, negativity was confirmed for neuroendocrine, neuroectodermal markers, NUT-1, MDM2, and their association with HPV and EBV.

Detailed histological analysis for each case was focused on selecting tumor areas with basaloid or high-grade characteristics, that were distanced from inflammation, hemorrhage, or necrosis, to avoid alterations in immunohistochemical assay expression. The concordance index (Fleiss’ Kappa 0.98) was obtained through interobserver measurements (two pathologist with head and neck pathology experience) for each carcinoma group analyzed.

Microarrays of tissue (TMAs) were then prepared following an established method [13]. Briefly, this technique involved creating an adhesive grid that was pre-sized to fit the histological embedding mold. Using a 3 mm diameter “punch” biopsy instrument, two tissue cylinders were extracted from the donor block and directly inserted onto the adhesive surface of the grid. Once the cylinders were arranged, they were immersed in a mold with molten paraffin at 70 °C until completely covered. The paraffin block containing the TMA was allowed to cool to −3 °C for 15 min. After the paraffin solidified, the grid was removed, and blocks were obtained; from these, 5-micron-thick sections were cut for evaluation with hematoxylin and eosin staining.

The inclusion criteria focused on the pathological parameters assessed in the SNSCC samples, namely evident squamous differentiation with or without transitional-like morphology, histological subtype, pattern of invasion, the imperative presence of high-grade characteristics (basaloid, rhabdoid, or plasmacytoid cytomorphology of tumor cells; high mitosis rate; nuclear and cytoplasmatic pleomorphism; and tumoral necrosis), vascular and neural invasion, tumor size, and extension to adjacent anatomical structures.

### 2.3. Immunohistochemical Assay for SWI/SNF Subunit Complex Evaluation

Immunohistochemical assays for SMARCB1 (clone MRQ27, Cell Marque, Rocklin, CA,USA; dilution 1:100) and SMARCA4 (clone BSB-154, BioSB, Santa Barbara, CA, USA; prediluted) were performed on 5-micron-thick sections following the standardized protocol on the Ventana Benchmark XT autostainer. The control tissue for the SMARCB1 antibody was the tonsil, and endothelial cells were evaluated as an internal control. For the SMARCA4 antibody, a germ cell tumor was used as the external control, while endothelial cells were used as the internal control.

Two methods were employed for result analysis. The first method utilized was the semi-quantitative Allred scale [14], which assesses both the intensity and percentage of positive cells in the sample. This scale consists of 8 points, derived from the sum of 6 ranges for positive cell counts (0 to 5) and 4 ranges for intensity (0 to 3). For our study, the samples were categorized into three groups based on the expression levels of SMARCB1/INI1 and SMARCA4/BRG1 according to the Allred score—expression levels between 0 and 2 were classified as “deficient”, scores of 3–5 were classified as “partial loss or mosaic expression”, and scores of 6–8 were classified as “intact” (Figure S1, Supplementary Material). The second method involved a quantitative analysis of the immunohistochemistry results using ImageJ^®^ software version 1.54 [15] (NIH, Bethesda, MD, USA). This analysis was performed on 5 photomicrographs at 400× magnification for each case, using the LEICA DM500 microscope equipped with the LEICA ICC50 W camera. Following software calibration, optical density (OpDe) measurements were taken to quantify the loss of SMARCB1 and SMARCA4 expression across 4 groups—absent (loss): 0–0.9; mild expression: 1–1.9; moderate expression: 2–2.9; and intense expression: >3 OpDe. For interpretation, 200 cells per photomicrograph were quantified to obtain the average optical density.

### 2.4. Follow-Up and Survival Analysis of SWI/SNF Partial Inactivation in Sinonasal Squamous Cell Carcinoma

Survival analysis was conducted using SPSS software version 30.0.0. The association between the partial loss of SWI/SNF complex subunits INI1/SMARCB1 and BRG1/SMARCA4 and time to death was evaluated using a multivariated test. Survival analysis was further performed using the Kaplan–Meier method and the log-rank test. A *p*-value of <0.05 was considered statistically significant.

## 3. Results

### 3.1. High-Grade Characteristics in Squamous Cell Carcinoma of the Paranasal Sinuses and Nasal Cavity

**Figure 1:**
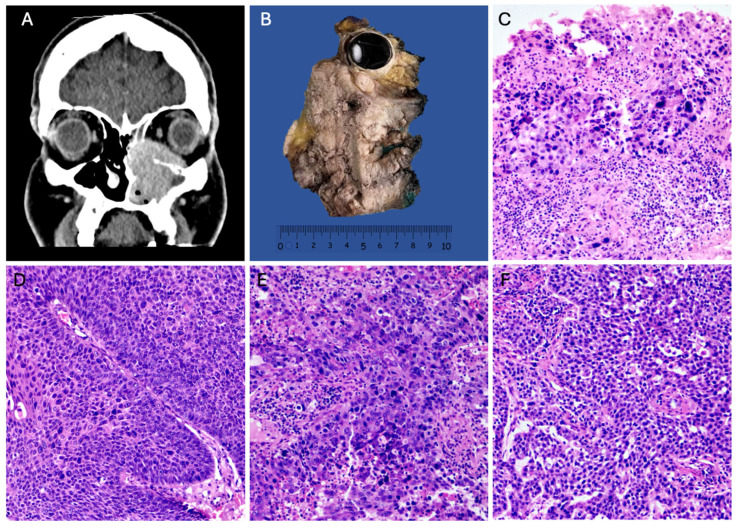
High-grade squamous cell carcinomas. (**A**) CT scan of a KSCC originating in the maxillary sinus with nasal and ethmoidal extension. (**B**) Gross image of a maxillectomy with orbital exenteration of KSCC. (**C**) HE 100× photomicrograph of SMARCA4-deficient sinonasal carcinoma. (**D**) HE 100× of NKSCC with high-grade features. (**E**) HE 100× of SCC with high-grade features. (**F**) HE 100× of SMARCB1-deficient sinonasal carcinoma.

A total of 103 cases of SNSCC with evident areas of high-grade transformation were collected over a 12-year period and were analyzed and subclassified as “high-grade squamous cell carcinomas”, characterized by basaloid, plasmacytoid, or anaplastic cellular features (Figure 1D,E). The distribution by gender revealed a higher incidence in males (59%) compared to females (41%) (Table 1). The age of presentation ranged from the second to the eighth decade of life, with a mean age of 54.1 years. High-grade sinonasal squamous cell carcinoma (NKSCC) was present in 47% of the cases (n = 48) (Figure 1D), while high-grade keratinizing squamous cell carcinoma (KSCC) represented 40% (n = 41) (Figure 1E). Nine cases exhibited a loss of SMARCB1 expression (Figure 1F), and 4% (n = 5) showed a loss of SMARCA4 expression (Figure 1C). The average tumor size for both squamous cell carcinoma groups was 7 cm (Figure 1A,B). Squamous cell carcinomas demonstrated a higher frequency of local invasion and locoregional lymphatic metastasis. The survival for the NKSCC group was 18.3 ± 16.08 months, while the KSCC group had a survival of 16.95 ± 17.04 months.

### 3.2. Partial Loss (Mosaic Expresion) of INI1/SMARCB1 and BRG1/SMARCA4 in Sinonasal Non-Keratinizing Squamous Cell Carcinoma

**Figure 2:**
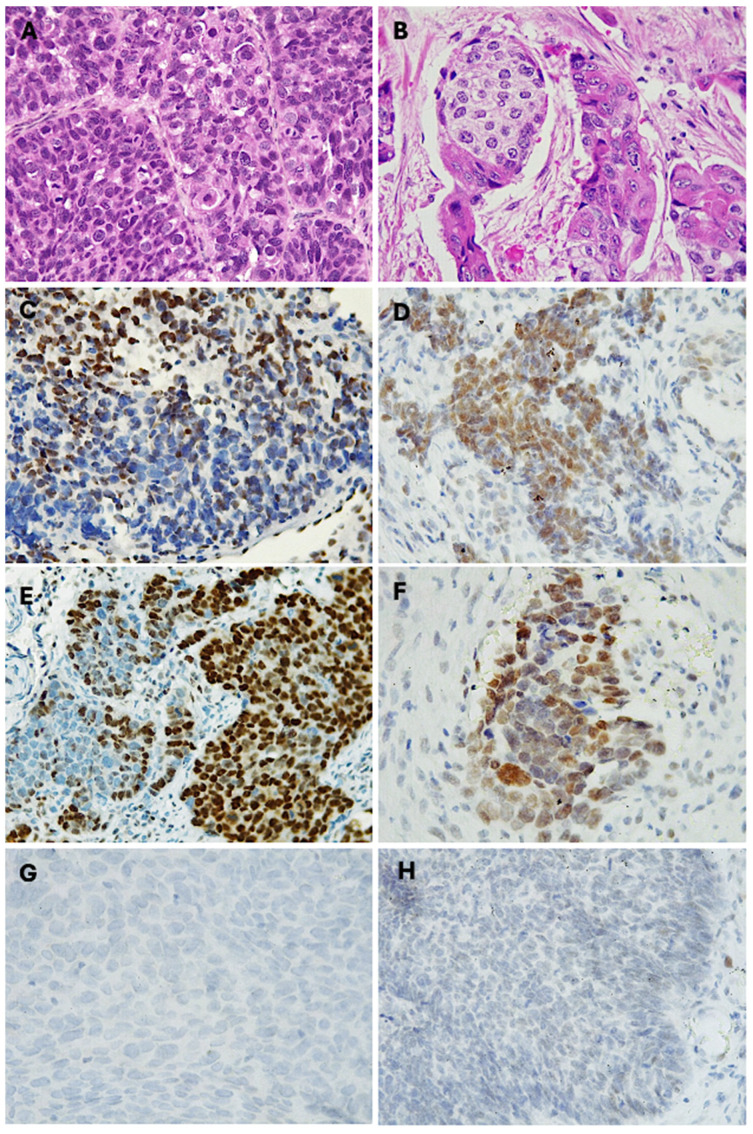
Partial loss of SWI/SNF complex in sinonasal SCC. Photomicrographs with hematoxylin and eosin-stained sections of (**A**) sinonasal non-keratinizing squamous cell carcinoma (400×), (**B**) sinonasal keratinizing squamous cell carcinoma (400×), (**C**) immunohistochemical mosaic expression of SMARCB1 in NKSSC (400×), (**D**) immunohistochemical mosaic expression of SMARCA4 in NKSSC (400×), (**E**) immunohistochemical mosaic expression of SMARCB1 in KSSC (400×), (**F**) immunohistochemical mosaic expression of SMARCA4 in KSSC, (**G**) SMARCB1-deficient sinonasal carcinoma, (**H**) SMARCA4-deficient sinonasal carcinoma.

Of the 48 sinonasal non-keratinizing squamous cell carcinoma (NKSCC) cases analyzed, 33.3% (*n* = 16) exhibited a mosaic expression pattern for INI1/SMARCB1. The loss of expression was particularly evident in the poorly differentiated high-grade tumor areas (Figure 2A). Cells lacking expression alternated with cells showing intact expression; thus, the pattern was classified as “mosaic expression” (Figure 2C).

Patients with a partial loss of SMARCB1 were predominantly male (56%) and female (44%), with a mean age of 48 ± 18.4 years. Tumor size averaged 5.0 ± 1.4 cm. The most affected anatomical site was the maxillary sinus (62.5%), followed by the nasal cavity (25%) and the ethmoid sinus (12.5%). Notably, 90% of the cases demonstrated invasion into adjacent anatomical structures and regional lymphatic metastasis, while 10% exhibited multiorgan metastasis (multicentric CNS dissemination, meningeal extension, and lung infiltration). At diagnosis, 75% of the cases were staged as IV (*n* = 12), and 25% as stage III (n = 4) (Table 1).

Among the 48 cases in the NKSCC group, 14% exhibited a decrease in BRG1/SMARCA4 expression. SMARCA4 showed partial mosaic inactivation, particularly in the anaplastic areas of the tumor located at the invasion front. A notable feature of the loss of expression was the gradual decrease in intensity in the tumor cells, indicating that the “intact” nuclear expression ranged from 67.83 to 94.36 OpDe, while the completely negative (deficient) cells ranged from 121.23 to 130 OpDe. (Figure 2D,F). The partial inactivation of SMARCA4 was more prevalent in males, comprising 57% of cases, compared to 43% (*n* = 3) in females. The average age was 48 ± 18.4 years, and the mean tumor size was 5 ± 1.5 cm. The most common primary tumor site was the maxillary sinus (57%), followed by the equal involvement of the nasal cavity, ethmoid sinuses, and frontal sinus, with one case (14.3%) each. In this group, 43% of carcinomas invaded adjacent structures such as the paranasal sinuses, orbit, and skull base. Three cases (43%) presented locoregional lymphatic and distant metastasis to the CNS and lung. Only one case (14%) showed isolated lymphatic metastasis. At the time of diagnosis, the oncologic staging of the patients was III and IV, with 29% in stage III and 71% in stage IV (Table 2).

### 3.3. Partial Loss (Mosaic Expresion) of INI1/SMARCB1 and BRG1/SMARCA4 in Sinonasal Keratinizing Squamous Cell Carcinoma

Of the 41 KSCC cases analyzed, 21% exhibited a mosaic expression pattern for INI1/SMARCB1. The loss of expression was particularly evident in basaloid cells with scant cytoplasm. The mosaic expression appeared in areas with limited keratin formation and primitive cytomorphology (Figure 2B,E). The partial inactivation of INI1 was slightly more prevalent in men, comprising 55% of cases, compared to 45% in women. The average age was 51 ± 11.5 years, with a mean tumor size of 7.8 ± 5.8 cm. The most affected anatomical site was the maxillary sinus (77.8%), followed by the nasal cavity and ethmoid sinus with 11.1%. In terms of metastasis, 44.4% of the cases showed lymph node involvement, while 44.4% had local invasion into adjacent anatomical structures such as the oral cavity and orbit. Only one case (11.1%) demonstrated metastasis to the C3 cervical vertebra. At the time of diagnosis, 55.6% of patients were in stage III, while 44.4% were diagnosed at stage IV (Table 2).

Of the 41 KSCC cases, 12.5% exhibited a reduced expression of BRG1/SMARCA4. The partial inactivation of BRG1 was identified as mosaic expression in areas with higher histological aggressiveness and at the tumor invasion front (Figure 2F). In this group of carcinomas, the partial inactivation of SMARCA4 was more prevalent in women (60%) than in men (40%). The average age of presentation was 61 ± 15.3 years, with a mean tumor size of 5.8 ± 2.4 cm. The most affected anatomical locations were the nasal cavity and maxillary sinus, each representing 40% of the cases. The frontal sinus was affected in one case, accounting for 20% of the carcinomas with altered SMARCA4 expression. Locoregional lymph node metastases were observed in 40% of the cases. Another 40% presented locoregional lymph node metastasis with invasion into other structures, with one case extending into the orbit and another into the skull base with meningeal extension. Only one case (20%) presented regional lymph node metastasis with distant spread to the lumbar vertebrae (Table 2).

### 3.4. Multivariated and Survival Analysis of SNSCC with Partial Inactivation of SWI/SNF Complex

**Figure 3:**
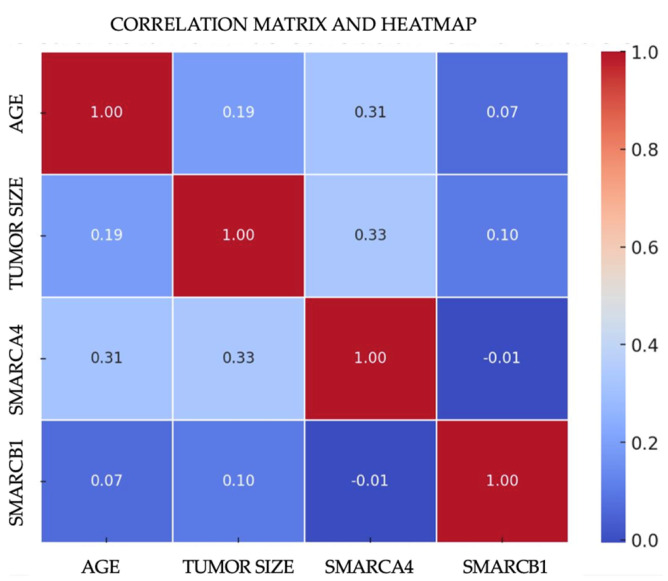
Heatmap correlation of mosaic SWI/SNF complex subunit expression with age and tumor size.

The multivariate analysis was conducted for both carcinoma groups (NKSCC and KSCC) through Pearson correlation studies to evaluate the following variables: tumor size, age, and mosaic expression of SMARCB1 and SMARCA4. In the NKSCC group, it was found that the mosaic expression of SMARCA4 and the age at tumor presentation (*p* = 0.029) suggest a significant and intriguing correlation that is more prevalent among tumors that developed between the fourth and fifth decades of life. Regarding tumor size and SMARCA4 mosaic expression, a correlation was identified (*p* = 0.021), indicating a statistically significant relationship where tumors displaying a mosaic expression of SMARCA4 are considerably larger in size compared to the other carcinomas studied (Figure 3). Through ANOVA, it was determined that sex does not have a significant impact on the mosaic expression of SMARCA4 or SMARCB1. For the KSCC group, no significant correlation was identified between mosaic expression and intact SWI/SNF complex carcinomas.

**Figure 4:**
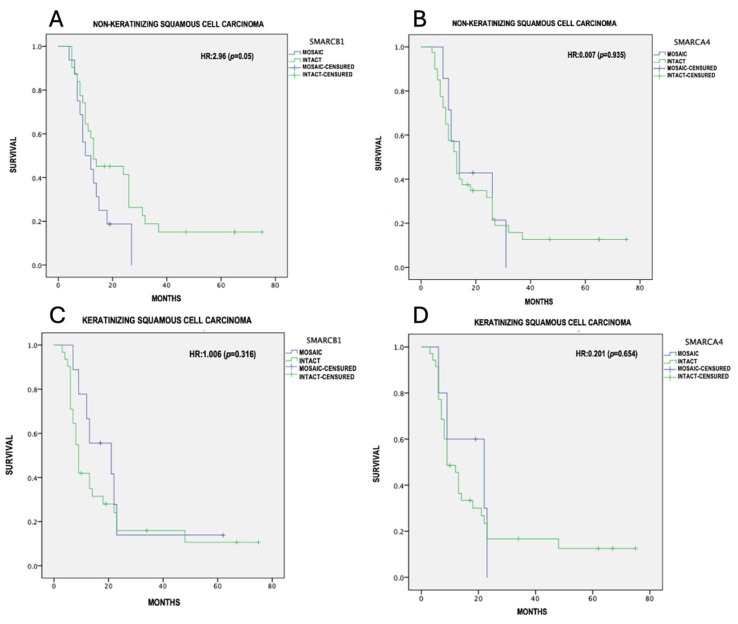
Survival analysis (Kaplan–Meier). (**A**) SMARCB1 mosaic expression in NKSCC, (**B**) SMARCA4 mosaic expression in NKSCC, (**C**) SMARCB1 mosaic expression in KSCC, (**D**) SMARCA4 mosaic expression in KSCC.

The overall survival was calculated using the Kaplan–Meier method with Cox analysis; differences in survival between mosaic and intact expression were calculated with the log-rank test, considering a *p* ≤ 0.05 as statistically significant. (Table 3)The mean survival was 18.3 ± 16.05 months for the NKSCC group, and 16.9 ± 17.04 months for the KSCC group. From the 48 NKSCC cases, only 4 were registered alive and disease-free at the 5-year follow-up. The remaining patients died due to tumor-related causes. In contrast to the KSCC group, only 6 out of 41 cases were alive and disease-free at the 5-year follow-up. The mean survival for the NKSCC group with the partial loss of SMARCB1 and SMARCA4 was 13.3 ± 1.9 and 18.3 ± 3.7 months, respectively. For the KSCC group with the partial loss of SMARCB1 and SMARCA4, the mean survival was 22.3 ± 6 and 16.5 ± 3.8 months, respectively. In our tumor-adjusted model, NKSCC showed an increased risk with the presence of SMARCB1 mosaic expression (HR = 2.96, 95% CI = 9.53–17.09, *p* 0.05), with a median survival of 13.31 months, compared to 25.0 months for intact expression (Figure 4A). NKSCC with the partial loss of SMARCA4 (HR = 0.007, *p* 0.93) (Figure 4B), KSCC with the partial loss of SMARCB1 (HR = 1.0, *p* 0.31) (Figure 4C), and KSCC with the partial loss of SMARCA4 (HR = 0.20, *p* 0.65) (Figure 4D) did not show statistically significant differences in survival.

## 4. Discussion

Sinonasal carcinomas are a group of tumors with locally aggressive behavior and an unfavorable prognosis due to their high rate of recurrence and progression and their capacity to generate metastases [5]. The genomic and epigenetic characterization of these tumors has allowed for their more precise classification [5,16].

The WHO classifies carcinomas originating in the paranasal sinuses, nasal cavity, and the skull base based on their histological appearance and genetic alterations into different groups—squamous cell carcinomas, lymphoepithelial carcinoma, HR HPV-related carcinomas (multiphase and monophase), NUT rearranged carcinomas, undifferentiated carcinomas (SNUC), neuroendocrine carcinomas, adenocarcinomas, and SWI/SNF complex-deficient carcinomas [16,17].

Squamous cell carcinomas originating in the sinonasal tract are rare neoplasms with a high rate of progression. The risk factors for the development of these neoplasms include chronic exposure to wood dust, industrial materials, smoking, human papillomavirus infections, and oncocytic sinonasal papillomas [5,7]. In our study, SNSCCs accounted for 64% of all tumors that developed in the sinonasal tract over a 12-year period, which is consistent with previous reports [7,18]. We found that some patients with SNSCC exhibited risk factors such as high-risk HPV exposure, exposure to wood smoke, and industrial materials [18,19,20].

The inadequate functioning or inactivation of the SWI/SNF complex can lead to distinctive cellular morphological changes such as rhabdoid and plasmacytoid morphology, large anaplastic cells, or small round cells in various soft tissue neoplasms and carcinomas affecting multiple organs [21,22,23,24,25]. A wide variety of benign and malignant tumors with alterations in several genes belonging to the SWI/SNF complex have been described, including SMARCB1, SMARCA4, SMARCA2, and ARID1A. These related tumors affect a range of tissues, primarily including epithelial, meningeal, glioneural, hematologic, and lymphoid tissues [26,27].

SMARCB1 (INI1)-deficient sinonasal carcinomas represent a group of poorly differentiated carcinomas characterized by the loss or complete inactivation of the SMARCB1 subunit, resulting in a basal-like, anaplastic, and even rhabdoid morphology. In our study, we identified nine SMARCB1-deficient carcinomas, of which 77% were men and 23% were women, which is a similar distribution to previous case series reported [28]. The aggressiveness of this type of tumor was measured based on the high mortality rate with a median survival of 5 months after diagnosis in our cases. Additionally, there was a high frequency of locoregional disease and distant organ involvement, consistent with previously reported findings [28].

In addition to the “loss expression” due to the complete genetic inactivation of the SWI/SNF complex subunits, a pattern of partial expression or “mosaic expression” has been recognized in conditions such as schwannomatosis and tumors such as gastrointestinal stromal tumor (GIST) and ossifying fibromyxoid tumor [29,30]. However, there are no reports of mosaic expression or the partial inactivation of the SWI/SNF complex in carcinomas affecting the sinonasal tract. Only a complete loss of expression, assessed by immunohistochemistry of SMARCB1 and SMARCA4, has been reported in sinonasal tumors [30,31].

In this study, we focused on sinonasal squamous cell carcinoma (SNSCC) for the following reasons: (1) it is the most common type of tumor that develops in the paranasal sinuses and nasal cavity; (2) it has a broad morphological spectrum—despite being classified into two histological subgroups (keratinizing and non-keratinizing), it can exhibit a variety of growth patterns such as papillary, basal-like, exophytic, infiltrative, acantholytic, and sarcomatoid (spindle cells); and (3) its morphological heterogeneity also results from a wide range of processes, pathways, and molecular alterations involved in its origin and progression, making it susceptible to various genetic and transcriptional alterations that influence its biological behavior and prognosis [31,32].

We included a total of 89 SNSCCs with high-grade morphological characteristics, where 33% of the NKSCCs and 21.9% of the KSCCs exhibited mosaic expression for SMARCB1. When evaluating pathological features and follow-up, differences were identified between carcinomas with intact expression and those that were deficient. One notable parameter was the average tumor size, which was similar between both groups with an average of 5 cm, compared to the 7 cm average reported for SMARCB1-deficient carcinomas [28]. Kaplan–Meier survival analysis showed a relationship between SMARCB1 mosaic expression in NKSCCs and prognosis, with a more unfavorable outcome compared to KSCCs with mosaic expression (SMARCB1 mosaic expression HR = 2.9, *p* 0.05).

Deficiency in the SMARCA4 subunit has been described in rare malignant neoplasms with poor prognosis, such as small cell carcinoma of the ovary, hypercalcemia type (SCCOHT), some thoracic carcinomas, gastrointestinal and genitourinary tract carcinomas, and non-small-cell lung carcinoma [33]. In the sinonasal tract, primarily two neoplasms deficient in SMARCA4 have been reported—SMARCA4-deficient sinonasal carcinoma and SMARCA4-deficient sinonasal teratocarcinosarcoma [6,33,34,35]. In the present study, we analyzed the SMARCA4 status in SNSCCs, finding mosaic expression in 14% of high-grade malignancy SN-NKSCCs and in 12% of keratinizing SNSCCs.

SMARCA4-deficient sinonasal carcinomas are a type of poorly differentiated or undifferentiated tumor, which are locally very aggressive and have a poor short-term prognosis—these are characteristics shared with sinonasal undifferentiated carcinoma (SNUC). One distinguishing difference between this group of lethal carcinomas and SMARCA4-deficient sinonasal carcinomas is IDH2 mutation [36,37,38].

The mosaic expression of SMARCA4 has not been reported in tumors as extensively as the mosaic expression of SMARCB1. This study offers an opportunity to assess its impact on other neoplasms in the sinonasal tract or other organs with potential SWI/SNF complex alterations. Current genetic studies and clinical trials are increasingly focused on identifying targets for classification, risk assessment, and therapeutic strategies, particularly in high-morbidity and mortality neoplasms like sinonasal carcinomas [39]. We believe the findings presented in this study significantly contribute to the understanding of sinonasal carcinomas with SWI/SNF complex alterations. Furthermore, our results may pave the way for future studies that explore the diagnostic and prognostic value of SMARCB1 and SMARCA4 mosaic expression in classification and therapeutic planning.

## 5. Conclusions

SWI/SNF complex-deficient sinonasal carcinomas are now well recognized as high-grade malignancies with distinctive deficiencies in the SMARCB1 and SMARCA4 subunits, distinguishing them from other neoplasms such as SNUC or poorly differentiated carcinomas. The mosaic expression of SMARCB1 has been reported in soft tissue tumors as a diagnostic tool and a parameter of morphological aggressiveness. Based on morphological, immunohistochemical, and statistical grounds, we conclude that the partial loss (mosaic expression) of SMARCB1 in SNSCC is associated with high-grade malignant characteristics and a negative effect on patient survival; meanwhile, SMARCA4-mosaic expression in SNSCC is associated with high-grade malignant characteristics and an increase in tumor size concerning the intact SMARCA4.

Comparing the mosaic expression of SMARCA4 versus SMARCB1, we observed a difference in the intensity of expression, whereby the mosaic immunohistochemical reaction for SMARCB1 was intense in the intact cells and completely absent in SMARCB1-deficient cells as expected, whereas SMARCA4-mosaic expression showed a gradual loss of expression from intensely positive (SMARCA4-intact cells) to those cells with the complete absence “loss” of expression. This suggests that the inactivation of these two complex subunits occurs in a gradual process that impacts the morphology during the cell de-differentiation process.

## Figures and Tables

**Table 1:** SWI/SNF complex expression in sinonasal carcinomas.

	Age (Mean Years)	Sex	Tumor Size (Mean cm)	SWI/SNF Complex (SMARCB1/SMARCA4)	Metastasis	Outcome
SN-NKSCC *n* = 48	55 years	M: 56% F: 44%	7 ± 3.9 cm	SMARCB1-intact: 67%	Local invasion: 43.7%	DWD: 81%
				SMARCB1-mosaic: 33%	Lymph node: 29.1%	LWD: 8.3%
				SMARCA4-intact: 85.4%	Multiorgan: 6.2%	DFD: 2.4%
				SMARCA4-mosaic: 4.6%	Local and distant: 21%	LFD: 8.3%
SN-KSCC *n* = 41	57.9 years	M: 63% F: 37%	7.2 ± 4 cm	SMARCB1-intact: 78.1%	Local invasion: 48.7%	DWD: 80%
				SMARCB1-mosaic: 21.9%	Lymph node: 31.7%	LWD: 2.2%
				SMARCA4-intct: 87.8%	Multiorgan: 2.4%	DFD: 2.2%
				SMARCA4-mosaic:12.2%	Local and distant: 17.2%	LFD: 14.6%
SMARCB1-deficient carcinoma *n* = 9	53 years	M: 77.7% F: 22.3%	6.7 ± 3.3 cm	SMARCB1-deficient: 100%	Local invasion: 44.5% Lymph node: 11% Local and distant: 44.5%	DWD: 90% LFD: 10%
SMARCA4-deficient carcinoma *n* = 5	65.5 years	M: 80% F: 20%	8.7 ± 1.9 cm	SMARCA4-deficient: 100%	Lymph node: 80% Local and distant metastasis: 20%	DWD: 80% DFD: 20%

M: male; F: female; DWD: death with disease; LWD: live with disease; DFD: death free of disease; LFD: live free of disease.

**Table 2:** Sinonasal squamous cell carcinoma with partial loss of SMARCB1 and SAMRCA4.

	SWI/SNF Complex Mosaic Expression	Age (Mean Years)	Sex	Tumor Size (Mean cm)	Anatomical Site	Metastasis	Oncologic Stage
Sinonasal Non-Keratinizing Squamous Cell Carcinoma	SMARCB1/INI 1	52.1 ± 13.8	M: 56.2% F:43.8%	5.9 ± 2.7	Maxillary sinus: 62.5% Ethmoid sinus: 12.5% Nasal cavity: 25%	Local invasion: 31.3% Lymph node: 31.3% Multiorgan: 6.3% Local and distant: 33.3%	DWD: 87.5% LWD: 12.5%
	SMARCA4/BRG1	48 ± 18.4	M: 57.1% F:42.9%	5.0 ± 1.4	Maxillary sinus:57%	Local invasion: 32.9%	DWD 85.7% LWD: 14.3%
					Ethmoid sinus:14.3%	Lymph node: 14.3%	
					Frontal sinus:14.3%	Multiorgan: 14.3%	
					Nasal cavity: 14.3%	Local and distant: 28.6%	
Sinonasal Keratinizing Squamous Cell Carcinoma	SMARCB1/INI 1	51 ± 11.5	M: 55.6% F: 44.4%	7.8 ± 5.8	Maxillary sinus: 77.8%	Local invasion: 44.5%	DWD: 67% LWD: 23% DFD: 10%
					Ethmoid sinus: 11.1%	Lymph node: 11%	
					Nasal cavity: 11.1%	Local and distant: 44.5%	
	SMARCA4/BRG1	61 ± 13.3	M: 40% F: 60%	5.8 ± 2.5	Maxillary sinus: 40%	Local invasion: 40%	DWD: 80% DFD: 20%
					Frontal sinus: 20%	Lymph node: 40%	
					Nasal cavity: 40%	Local and distant: 20%	

M: male; F: female; DWD: death with disease; LWD: live with disease; DFD: death free of disease.

**Table 3:** Mosaic expression in NKSCC and KSCC (Cox analysis).

Tumor	SWI/SNF Complex	HR (Hazard Ratio)	IC 95%	*p*-Valor
Sinonasal Non-Keratinizing Squamous Cell Carcinoma	SMARCB1/INI 1	2.9	Intact: (16.6–33.3) Mosaic: (9.5–17.09)	*p* = 0.58
	SMARCA4/BRG1	0.007	Intact: (19.8–29.1) Mosaic: (10.9–25.7)	*p* = 0.007
Sinonasal Keratinizing Squamous Cell Carcinoma	SMARCB1/INI 1	1.006	Intact: (11.1–27.3) Mosaic: (10.5–34.1)	*p* = 0.316
	SMARCA4/BRG1	0.201	Intact: (12.6–28.2) Mosaic: (8.4–24)	*p* = 0.65

## Data Availability

The results are available on reasonable request from the correspondence of this study.

## Supplementary Materials

The following supporting information can be downloaded at: https://www.mdpi.com/article/10.3390/cimb46110723/s1, Figure S1: Immunohistochemical staining for SWI/SNF complex evaluation.
