# Supplementary material for: Switch/Sucrose Non-Fermentable (SWI/SNF) Complex—Partial Loss in Sinonasal Squamous Cell Carcinoma: A High-Grade Morphology Impact and Progression
Source: Curr Issues Mol Biol. 2024 Oct 30;46(11):12183–95. doi: 10.3390/cimb46110723 (PMC11592847; doi:10.3390/cimb46110723)
Supplement: Supplementary file 1 [file cimb-46-00723-s001.zip › cimb-3267125-supplementary.pdf]

Fig S1 (Supplementary Material)

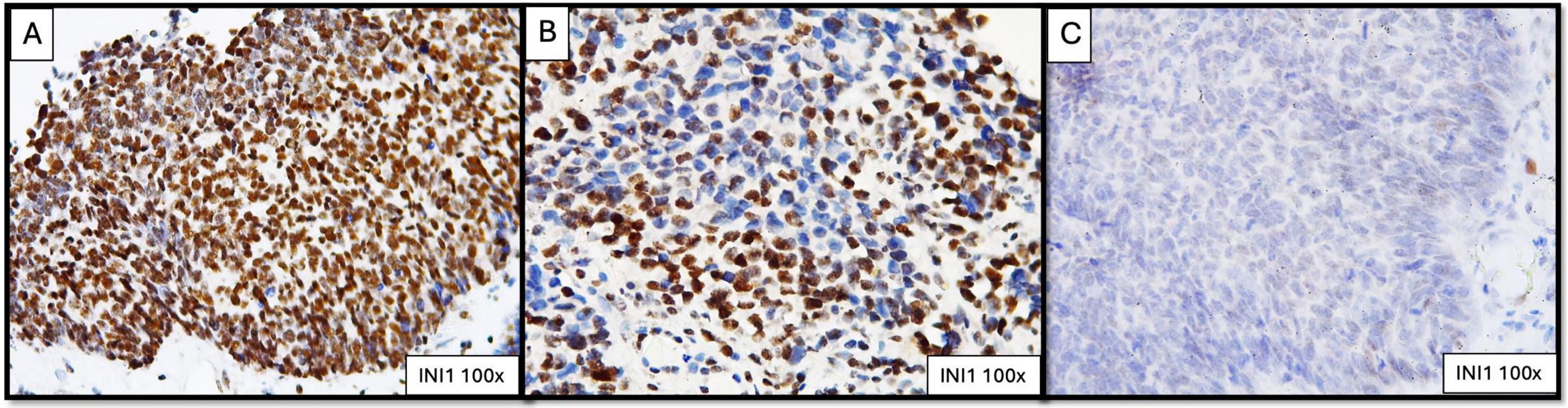

Figure S1. Immunohistochemical staining for SWI/SNF-complex evaluation. (A)" intact" expression with a diffuse staining in the neoplastic cells, (B) "mosaic" expression is presented with a patchy pattern. (C) Deficient expression with comprehensive negative cells in the tumor.
